# High-precision gas refractometer by comb-mode-resolved spectral interferometry

**DOI:** 10.1038/s41598-018-34641-y

**Published:** 2018-11-06

**Authors:** Lijun Yang, Yan Li, Haoyun Wei

**Affiliations:** Key Lab of Precision Measurement Technology & Instrument, Department of Precision Instrument, Tsinghua University, Beijing, 100084 China

## Abstract

High-accuracy knowledge of gas refractivity is typically crucial for optical interferometry, precise optical systems, and calculable pressure standard development. Here, we demonstrate an absolute gas refractometer by spectral interferometry and a high-resolution spectrometer. The spectral interferometry relies on a comb with fiber Fabry–Pérot filtering cavity, and a double-spaced vacuum cell. The spectrometer employs a virtually imaged phased array, diffraction grating and near-infrared camera to fully resolve the comb modes. Finally, by means of fast-Fourier-transform, the group refractivity can be derived from the spectrally resolved interferograms of the two beams propagating in the inside and outside of the vacuum cell. To confirm the feasibility and performance of the gas refractometer, the measurement of ambient air was conducted. The proposed scheme has a combined uncertainty of 1.3 × 10^−9^ for air and a single measurement only takes 10 ms, which is applicable for gas refractivity monitoring and compensating in real time.

## Introduction

High-precision refractive index of an ambient gas is of great importance in the following three aspects. First of all, optical interferometric metrology is mostly carried out in atmospheric conditions^1–6^, where the accuracy of air refractive index is a severe limitation to the best measurement uncertainty. For example, to reach the precision level of nanometer within hundreds of millimeters in precision instruments manufacturing, the value of air refractivity should be known with an accuracy of 10^−7^ or better. The second one is in the area of precise optical systems, such as projection photolithography objective lens. In order to reduce corrosion of the film layer on each lens by air, Nitrogen (N_2_) is usually used as a protective gas between the lenses. However, the N_2_ refractivity will vary with the changes of temperature, pressure and other parameters during the working process^7,8^, resulting in focus drift, magnification change, and distortion. Therefore, accurate Nitrogen refractivity measurement is of great significance to the design, adjustment and testing of objective lens. Furthermore, high precision gas refractivity measurement has great potential in realizing the unit of pressure at the highest level of metrology^9,10^. In 1998, Moldover first demonstrated the possibility of establishing the thermodynamic pressure standard based on ideal gas law^11^. According to the *ab initio* quantum calculations, since the theoretical accuracy of the density virial coefficient of helium (He) has exceeded the best experimental results, the relationship of refractivity to pressure with a high accuracy is only possible for He at present, due to the complexity of other atoms or molecules^12^. From the point of view of uncertainty analysis, a 10^−9^ uncertainty of He refractivity can achieve a 10^−6^ uncertainty of pressure up to three mega pascals^13^.

In view of the significance of gas refractivity mentioned above, various refractivity measurement methods have been developed. Air, as the most common gas, is conventionally calculated according to empirical equations using data of the environmental parameters^14–19^, with an uncertainty of (3–5) × 10^−8^. However, the results from empirical equations are not credible under circumstances where environment fluctuates rapidly or air components change. Consequently, to achieve *in-situ* monitoring or get better accuracy, direct measurements are then the optimal option. The direct methods for gas refractivity measurement are usually divided into two generic approaches. The first one is based on incremental interferometry by recording the interferometric phase change continuously during the procedure of gas filling or pumping^20–22^. Nevertheless, this kind of method is not only complicated but also time-consuming. Moreover, there will exist pressure and temperature fluctuations during the measurement. The second approach is called absolute measurement, which means it is needless to do successive phase measurement during the period of gas pumping or filling. Recently, some absolute methods for gas refractivity measurement have been reported by use of a Fabry–Pérot cavity^23^, two-color method^24–27^, trapezoidal cavity^28^, and synthetic pseudo-wavelength (SPW) method^29,30^. However, Fabry–Pérot cavity method also needs measurements under vacuum to obtain the ultimate value. Two-color method needs an initial value to get the actual refractivity. The trapezoidal cavity requires a high manufacturing precision to measure the refractivity accurately. As for the SPW method, to construct the SPW chain, the geometric lengths of the three used vacuum cells needs to meet the conditions of each wavelength grade.

In our former work, an absolute air refractive index measuring apparatus was proposed^31^. At that time, the dispersive interferograms were measured through a commercial OSA (optical spectrum analyzer). However, the wavelength resolution of the OSA is low (0.2 nm) and the spectral frequencies are inaccurate caused by frequency sampling nonlinearity, which contributes much to the measurement uncertainty of air refractive index. In this paper, we establish an absolute gas group refractometer by spectral interferometry and a high-resolution spectrometer. Here, the spectral interferograms are obtained by a high-resolution spectrometer consisting of a VIPA (virtually imaged phased array), diffraction grating and near-infrared camera^32^. The 250-MHz-spaced comb modes are too densely spaced to be resolved by the VIPA-based spectrometer, so we first use a FFP (fiber Fabry–Pérot) cavity with a FSR (free spectral range) of 5 GHz to filter out unwanted comb lines, generating a 2-dimensional (2D) array of well separated bright spots when imaged by the camera. Since every spot corresponds to the resolved frequency of one unique comb mode, the measurement uncertainty caused by the frequency sampling nonlinearity can be avoided. Besides, with this simple configuration, the refractometer becomes compact and promising for field applications.

## Measurement Principles

In our measurement scheme, the filtered output of a femtosecond optical frequency comb after a FFP cavity is sent into a Michelson interferometer. The spectral interferograms are then analyzed by a high-resolution spectrometer. In the spectrometer setup, the VIPA etalon is a primary component, whose spectral dispersion is perpendicular to that of one dispersion grating. The etalon is tilted slightly from normal incidence so that the beam transmitted from the VIPA resembles an elongated stripe of light. Then one diffraction grating is used to disperse the output of the VIPA in the horizontal direction. Next, the 2D mode-resolved image of the interferometer output is acquired by a near-infrared camera. While the spectral resolution of VIPA is roughly several tens fold higher than that of a normal dispersion grating^33^, the optical grating is used to separate different orders of light transmitted through the VIPA etalon horizontally. Therefore, the spectral resolution of the dispersion grating must be greater than the FSR of the VIPA under such condition. From the obtained spectrometer image where any individual spatial unit represents a comb mode frequency, a one-dimensional (1D) interferograms of respective interference intensity with frequency can be reconstructed by concatenating the spatial elements along a reasonable zigzag line.

In theory, the spectral interference information acquired from the 2D spectral image is expressed as follows^34^:

1 $$I(v)={I}_{0}(v)+1/2[{I}_{m}(v)\,\exp ({\rm{i}}\cdot \phi (v))+{I}_{m}(v)\,\exp (-{\rm{i}}\cdot \phi (v))],$$

where *I*(*v*) is interferometric optical intensity, i = (−1)^1/2^, *I*_0_(*v*) denotes the average intensity of the two interference light beams, *I*_*m*_(*v*) represents the modulation intensity, and *φ*(*v*) is the interferometric phase difference, which is:

2 $$\phi (v)=\frac{4\pi v(n(v)-1)L}{c},$$

where *L* indicates the length of the double-spaced cell. *n*(*v*) and c denote gas refractivity and light velocity in vacuum. The Fourier-transformation of *I*(*v*) is complex, which can be represented as:

3 $${\rm{\Gamma }}(t)=FT\{I(v)\}={{\rm{\Gamma }}}_{o}(t)+{{\rm{\Gamma }}}_{m}(t)\otimes \pi [\delta (t+\tau )+\delta (t-\tau )],$$

where τ denotes the interferometric optical delay and *δ*(*t*) denotes unit pulse equation. Γ_0_(*t*) and Γ_m_(*t*) are the Fourier transforms of *I*_0_(*v*) and *I*_m_(*v*). To obtain the exact value of τ, only the peak at τ is picked out through one digital bandpass (BP) filter. Next, the filtered result is inverse Fourier-transformed as *I*’(*v*). After that, *φ*(*v*) can be calculated from the following equation^35^:

4 $$\phi (v)=\arctan (\frac{{\rm{I}}{\rm{m}}\{{I}^{{\rm{^{\prime} }}}(v)\}}{{\rm{R}}{\rm{e}}\{{I}^{{\rm{^{\prime} }}}(v)\}}).$$

The first-order derivative of the unwrapped interferometric phase difference is calculated as:

5 $$\frac{d\phi (v)}{dv}=\frac{4\pi (N-1)L}{{\rm{c}}},$$

where N = *n*_*p*_(*v*) + (*dn*_*p*_(*v*)/*dv*)*v*. Here, N is the group gas refractivity determined by the central frequency of the optical source, and *n*_*p*_ is the phase refractive index of gas. Because the repetition frequency (*f*_*rep*_) and carrier-envelope offset frequency (*f*_*ceo*_) of the frequency comb are stabilized against the rubidium atomic clock, and the filtered comb modes are resolved spectrally to the level of an individual mode, an almost perfect frequency scale is available for determining the first-order derivative of the interferometric phase difference with frequency. As we can see from equation (5), if an accurate value of *L* is given in advance, (N − 1) can be calculated via the following formula:

6 $$({\rm{N}}-1)=(\frac{{\rm{c}}}{4\pi L})\frac{d\phi (v)}{dv}.$$

In the computing procedure of FFT (Fast Fourier Transform), the Hanning window is used to get the best result^36^. Besides, the calculated result has a limited range. The minimum measurable (N − 1) is ~3.7 × 10^−6^ (*L* = 396 mm) determined by c/(2*Lw*), where *w* is the threshold determined by the temporal width of Γ_m_(*t*)^34^. The maximum (N − 1) is calculated as 0.0375 from c/(4*PL*). Here, *P* is the separation between the two adjacent modes-5 GHz. As a result, the measurable scope of N starts from the beginning of 1.0000037 to the end of 1.0375. As far as we know, this measurement range contains nearly all of the common gases varying considerably with time.

## Results

**Figure 1:**
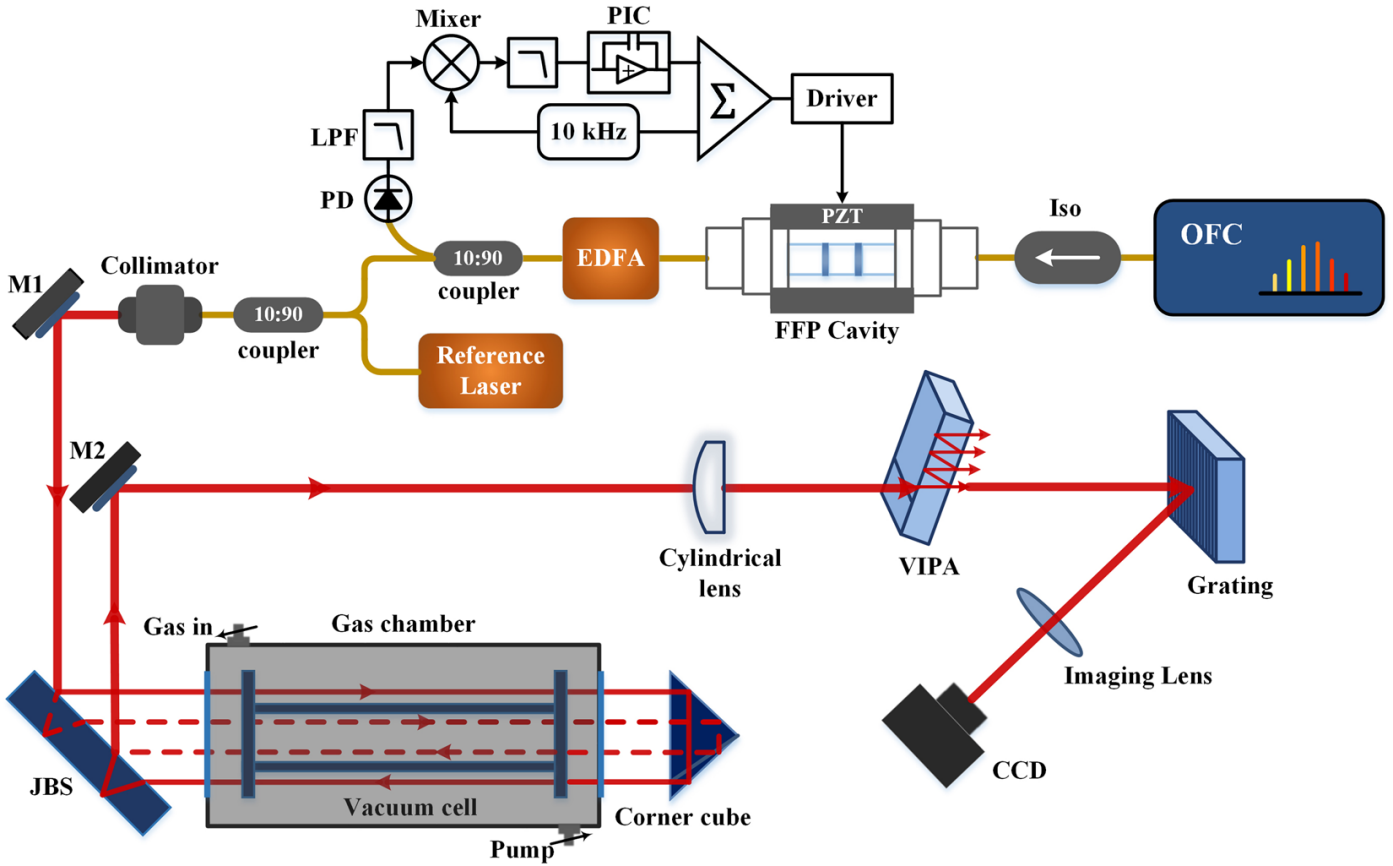
Experimental apparatus of the proposed gas refractometer. Iso: isolator; PD: photodetector; LPF: low-pass filter; M1, M2, mirror. The red line denotes the light beam propagating in the free space (the dotted line represents the light beam transmitting inside the vacuum cell), the yellow line illustrates the light beam passing through the optical fiber, and the black line denotes electrical connection.

The experimental apparatus is shown in Fig. 1. The light source is a femtosecond optical frequency comb (OFC, MenloSystems, FC1500), whose central wavelength and 3-dB bandwidth are around 1569.8 nm and 45 nm. Its *f*_*rep*_ and *f*_*ceo*_ are locked at 250 MHz and 20 MHz to a rubidium atomic clock (Symmetricom, 8040 C), separately. One tunable ECDL (external-cavity diode laser) (New Focus Velocity, TLB6728), as a reference to calibrate the comb mode frequency, is coupled into the same fiber that delivers the frequency comb light via a 10:90 fiber coupler. The collimated output of the combined optical source is incident to the Jamin beam splitter (JBS) at 45 degree. The size of JBS is specially designed so that the laser beam can be completely separated into two beams, and recombine again (size of JBS: 80 mm × 30 mm × 18.5 mm). Besides, the reflectivity of the upper and lower surface is 50% and > 99%, separately. The light beam is divided into two coaxial light beams through the JBS, whose light power are almost the same. After that, they propagate in the inside and outside of the fused silica vacuum cell, whose air pressure inside is lower than 0.01 Pa. Its window is coated with antireflective film and has a transmittance better than 99.8% within a range of 1510 nm-1610 nm. The actual length is measured as 396.000 mm by a CMM (Coordinate Measuring Machine) (HEXAGON Metrology, Optiv Reference 543) and its volume is about 1.24 × 10^5^ mm^3^. The vacuum cell is placed inside one gas chamber with two fused-silica windows. The gas to be measured can be filled in or pumped out via two pipes linked to the chamber. After reflected by the gold coated hollow corner cube, the two light beams come across again and interfere with each other on the upper surface of the JBS. The interference light is subsequently analyzed with a VIPA-based spectrometer. First, the interference light is line-focused with a cylindrical lens into the antireflection-coated access window of the VIPA (OP-6721–1686–8, Light Machinery), whose FSR is 60 GHz and finesse is ~110. Next, a 600 lines/mm diffraction grating (Thorlabs, GR25–0616) is used to disperse the light horizontally. With a 200 mm lens, the frequency-resolved image of the interferometric beam is recorded by one InGaAs camera (Xenics, Xeva-1.7–320), whose pixel size is 30 μm and read out time is about 10 ms. The integral time for the camera to capture one image is 100 µs and good fringe contrast images can be obtained for such acquisition time.

The theoretical resolution of the VIPA-based spectrometer is estimated to be ~1.5 GHz and it is remarkably larger than the comb mode separation. Therefore, to fully resolve the comb modes, the stabilized source-comb light passes through a FFP cavity to filter out unwanted comb lines and increase the line spacing. The FFP cavity is directly manufactured by optical fibers so that no alignment or mode-matching is required. The FSR of the cavity is set to 5 GHz (transmitting every 20th comb mode) because it is over three times the spectrometer resolution and can result in an optimal 2D spectrum without cross-talk^37^. The length of the FFP cavity is electronically stabilized to the highest intensity peak of the transmission via a dither-locking technique (refer to “methods” section for details). However, the transmitted optical power of the FFP cavity is low for subsequent experiment, so we use a homemade EDFA (erbium-doped fiber amplifier) to compensate the power loss induced by the filter cavity. After mode filtering and power amplifying, the central wavelength and 3-dB bandwidth of the comb are about 1530.8 nm and 37.5 nm. The 3-dB bandwidth is narrowed due to the existence of dispersion in the FFP cavity.

In order to reconstruct the spectral interferograms correctly, it is necessary to identify the frequency of individual imaged dots. Therefore, the frequency of the reference ECDL, *v*_*ref*_, is measured independently by a 50 MHz-accuracy wavemeter (Bristol 621), and the optical frequency of the nearest comb mode, *v*_*n*_, is deduced by limiting |*v*_*n*_ − *v*_*ref*_| in a range of 250 MHz, where: *v*_*n*_ = *n* × *f*_*rep*_ + *f*_*ceo*_ and *n* is the mode number. Here, *n* = int(*v*_*ref*_/*f*_*rep*_) (int means taking the integer portion of a digit number). The ECDL thus provides a known reference frequency to the VIPA-based spectrometer. Based on the reference marker and the values of *f*_*rep*_ and *f*_*ceo*_, the absolute frequency of every imaged dot can be determined.

### Image analysis and data processing

**Figure 2:**
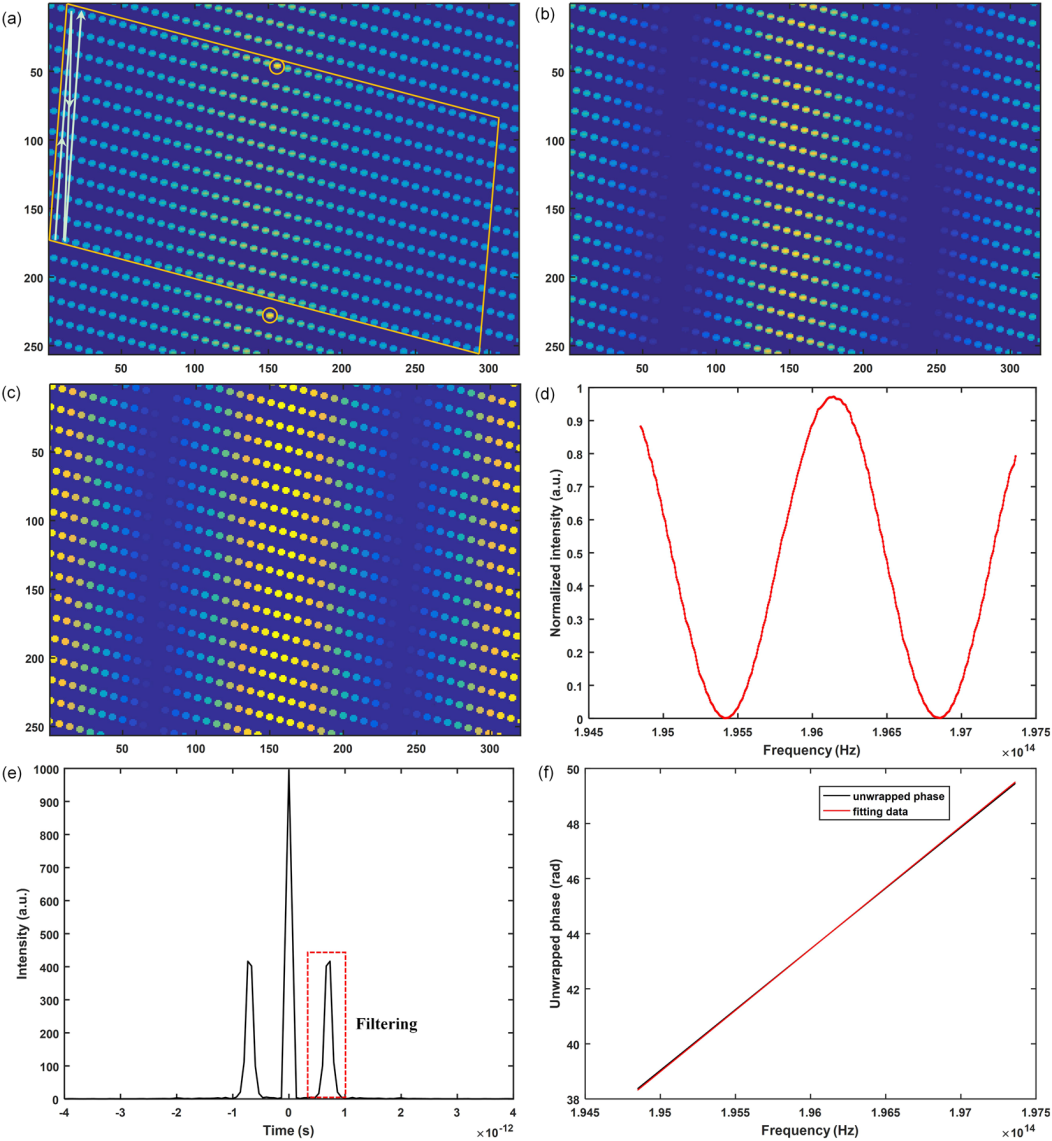
2D spectrogram and data computing process. (**a**) Camera image of filtered comb spectrum, 1D spectrum is reconstructed by stitching vertical lines inside the orange box together, as indicated with the white arrow. (**b**) Camera image of spectral interferogram at a certain moment. (**c**) Camera image normalized by filtered comb spectrum. (**d**) Reconstructed 1D spectra interferogram. (e) Fourier transform of the reconstructed spectral interferogram. (**f**) The unwrapped phase. In (**a**–**c**), yellow represents higher optical power.

In order to confirm the feasibility and performance of the gas refractometer, ambient air was measured using our refractometer. Figure 2(a) shows the camera image of the comb spectrum obtained from either the measurement or the reference arm individually. In Fig. 2(a), every image spot represents a unique resolved comb mode. The camera image of the spectral interferogram at a certain moment is shown in Fig. 2(b) and the brightness of every image spot depends on the interference phase difference. Figure 2(c) is the spectral interferogram that normalized by Fig. 2(a). In the perpendicular orientation of the three images, the information duplicates every 60 GHz at VIPA’s FSR, and a set of distinctive imaged spots are encircled in the orange box in Fig. 2(a). Inside the orange border, ~504 comb modes are apparently resolved, indicating that a bandwidth of 2.52 THz is acquired on the camera. The traditional 1D spectrum can be reconstructed by concatenating adjacent columns consisting of 12 spatial spots using the information of the calibration procedure by the reference ECDL. The output of the reference ECDL is imaged via the camera as two obviously brighter spots (represents two orders of the VIPA) orange circled in Fig. 2a. The measured frequency of ECDL by wavelength meter is 196098.2(88) GHz, thus the mode number of the more intense spot is 784393 and the respective frequency of all the modes inside the box can be known. To calculate the intensity of every imaged spot, a matched filter is used to the camera images. Specifically speaking, cross-correlations of the images are carried out with a 5 × 5 pixel kernel and the kernel is one imaged spot that resembles the profile of all resolved comb modes best^38^. Because the matched filter is the optimum linear technique to detect the existence of a signal mixed with white noise, the imaged spots that are only partially transmitted by the FFP cavity are easily removed from the camera image^37^. To eliminate the influence of common-mode brightness variations in the optical system, the reconstructed 1D spectral interferograms have been normalized to the envelope of the comb spectrum (Fig. 2(d)). Then we use FFT method to calculate the group air refractivity. The results derived from Ciddor’s equation^17^ are used to compare with our measured values.

During the period of our experiment, the pressure, temperature, CO_2_ concentration and humidity are monitored by corresponding sensors calibrated by the National Institute of Metrology of China: a 14 Pa-accuracy pressure gauge (Setra, Model 470), one 0.01 °C-accuracy thermometer (Fluke, 1523), a CO_2_ sensor with an accuracy of smaller than 50 ppm (TEL, 7001), and a hygrometer with an accuracy of no larger than 2% RH (Rotronic, HP23), respectively. To reflect the actual environmental parameters more accurately, the monitoring sensors are placed as close as possible to the center of the vacuum cell. Figure 2(e) shows the corresponding Fourier transform of the normalized spectral interferograms. The peak on the right is first selected by a BP filter and the inverse Fourier transform is carried out in the next step. The unwrapped phase is shown in Fig. 2(f) and its first-order derivative is 4.4515121820 × 10^−12^ rad/Hz. As a result, the group air refractivity is 1.0002683637 derived from equation (6). Compared with the computed values from *n*_*p*_ − (*dn*_*p*_/*d*λ)λ (*n*_*p*_ is obtained by the Ciddor’s equation) of 1.000268367 (temperature: 22.797 °C, pressure: 101.809 kPa, humidity: 28.24% RH, CO_2_ concentration: 1073 ppm), the difference is −3.3 × 10^−9^.

### Short-term stability

**Figure 3:**
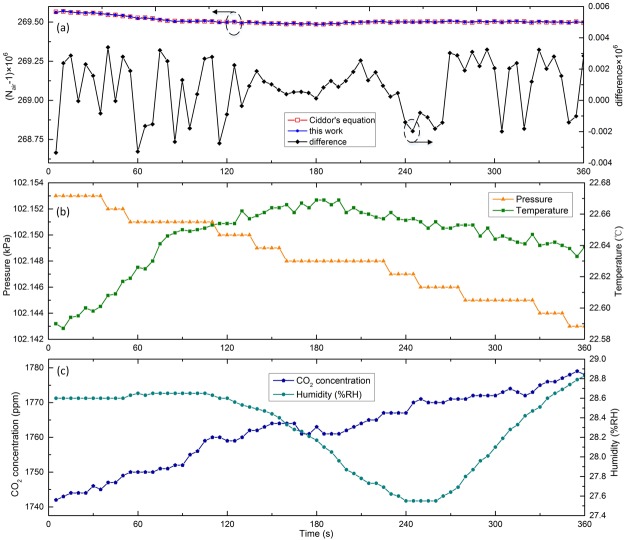
Short-term measurement data of group refractive index of ambient air. (**a**) Comparison between the measurement data and computed value from *n*_*p*_(*v*) + (*dn*_*p*_(*v*)/*dv*)*v* (*n*_*p*_ is obtained by the Ciddor’s equation). The arrow direction points out appropriate y axis. (**b**) Variations of pressure and temperature. (**c**) Variations of CO_2_ concentration and humidity.

To assess the accuracy and stability of the proposed scheme, air refractive index measurement was first performed in short time. In Fig. 3, continuous measurement of ambient air was carried out for 6 minutes (one single measurement was conducted every 5 seconds). It can be seen from Fig. 3(a) that the differences between the group air refractivity measured through our approach and those derived from Ciddor’s equation are less than 3.4 × 10^−9^, with a standard deviation of 1.8 × 10^−9^. As is shown in Fig. 3(b,c), the pressure, temperature, CO_2_ concentration and humidity changed by 10 Pa, 0.082 °C, 37 ppm, and 1.28% RH in the process of this measurement.

### Long-term performance

**Figure 4:**
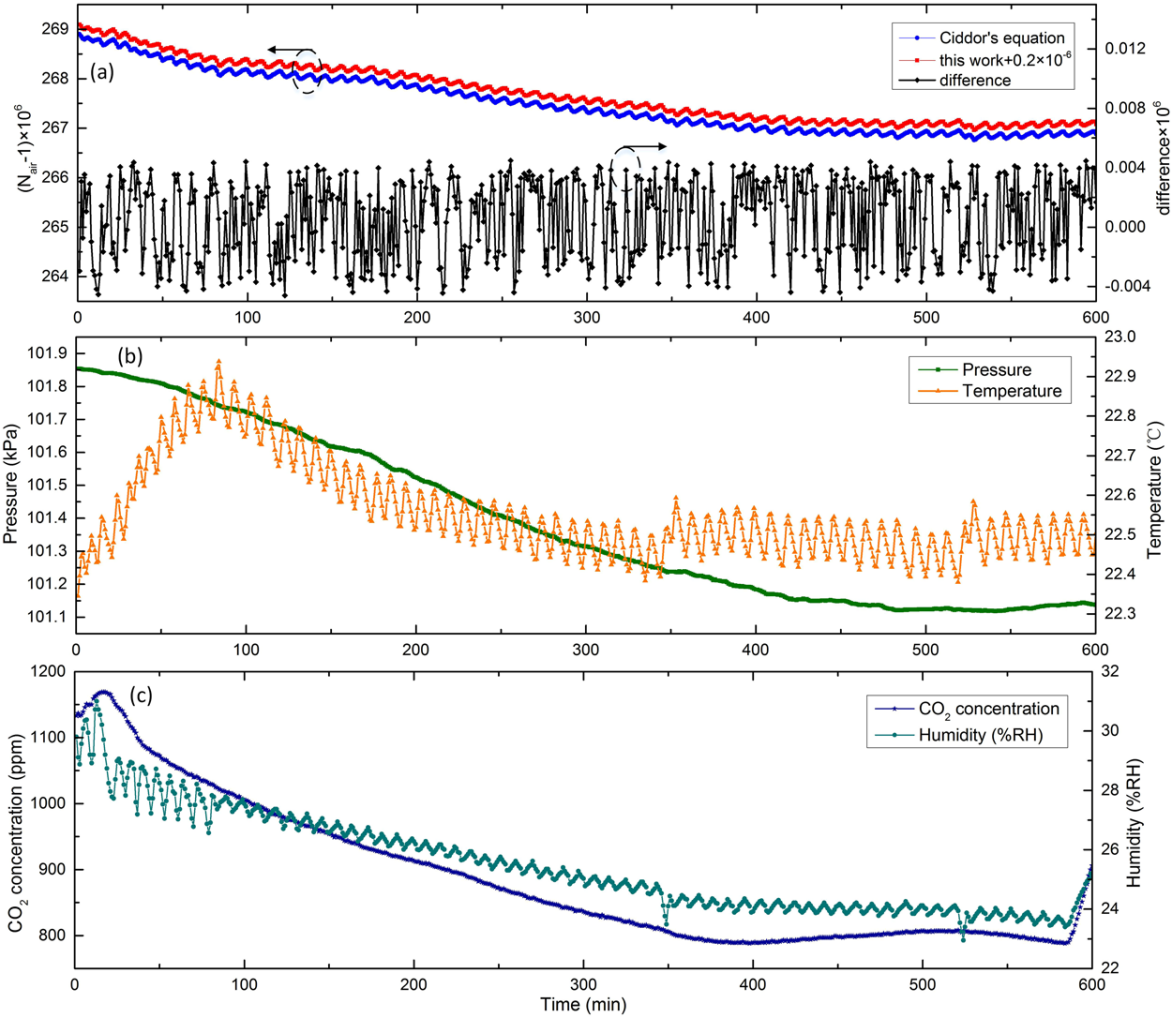
10-h measurement of the group air refractive index. (**a**) Comparison between the measurement data and computed value from *n*_*p*_(*v*) + (*dn*_*p*_(*v*)/*dv*)*v* (*n*_*p*_ is obtained by the Ciddor’s equation). The arrow direction points out appropriate y axis. (**b**) Variations of pressure and temperature. (**c**) Variations of CO_2_ concentration and humidity.

To assess the performance of the proposed scheme further, refractivity measurement of ambient air was also performed in long time. This successive measurement lasted ~10 hours (the time interval between two successive measurements was 1 minute) and Fig. 4 illustrates the measured results. We can find in Fig. 4(a) that the variation trends of measured data and computed results are almost identical. Their differences are less than 4.6 × 10^−9^ with a standard deviation of 2.7 × 10^−9^, which proves that the measurement apparatus performs high stability. Be aware that to separate these two curves, the measurement result is added by 2 × 10^−7^ for better visualization. It can be seen in Fig 4(b,c), during such long-term measurement, the pressure, temperature, CO_2_ concentration and humidity fluctuated by 736 Pa, 0.6 °C, 381 ppm and 8.05% RH, separately. Compared with the results in our former study^31^, the long-term measurement accuracy is increased by ~5 times, showing that the VIPA-based spectrometer is effective in improving the measurement accuracy.

### Uncertainty evaluation

In our experiment, frequency error, length error of the double-spaced cell, the interferometric phase difference error, and vacuum degree of the double-spaced cell are considered.

Since every comb mode is fully resolved by the VIPA-based spectrometer and the absolute frequency of every imaged dot can be determined on the basis of the reference ECDL and the values of *f*_*rep*_ and *f*_*ceo*_, this error source is caused by the frequency stability of the optical comb. During the period of measurement, both *f*_*rep*_ and *f*_*ceo*_ are phase-locked against a rubidium atomic clock, thus the frequencies of the comb modes have a stability comparable to the stability of the reference clock. As for the used rubidium atomic clock, this is 2.4 × 10^−11^ in 1 second. Therefore, the frequency error contributes ~2.4 × 10^−9^ in 10 ms to the relative measurement uncertainty of (N − 1) and can be neglected.

The measured length of the double-spaced cell by the CMM has an accuracy of about 0.5 μm. From the length error analysis of vacuum cell in our previous work^31,39^, the path difference caused by the non-coaxiality between the vacuum cell and the optical axis is less than 0.2 μm. The maximum possible temperature variation introduced error is 0.6 μm. Besides, window deformation caused by atmospheric pressure difference is 0.56 μm. Above all, the entire length error provides ∼4.7 × 10^−6^ to the relative measurement uncertainty of (N − 1).

The interferometric phase difference is computed via FFT algorithm and the obtained data is mainly affected by two items: the intensity accuracy of the spectral interferograms, and the computing accuracy of the adopted FFT algorithm. Since the spectral interferograms are acquired by an InGaAs camera with a 12-bit resolution, the intensity measurement accuracy is 1/2^12^, resulting in an equal size of error with that of the interferometric phase difference and accounts for 2.7 × 10^−7^ to the relative uncertainty of (N − 1). Besides, because the reconstructed spectral interferograms have been normalized to the envelope of the comb spectrum, the common-mode intensity noise can be eliminated. From our previous work^31^, the calculated accuracy of the adopted FFT algorithm is ~1 × 10^−9^. Moreover, the non-ideal glass or fused silica optics and their inhomogeneity also affect the accuracy of the interferometric phase difference. To eliminate their influence, we first evacuated the gas chamber to vacuum using a mechanical vacuum pump and a turbomolecular vacuum pump, and then we measured the existed interferometric phase difference. When we measured the gas refractive index, the phase difference caused by the non-ideal glass or fused silica optics were subtracted from the total interferometric phase difference, thus this error can be eliminated. In total, the phase difference error shares 2.8 × 10^−7^ to the relative uncertainty of (N − 1).

From Equation (6), we know that N = 1 + (*dφ*(*v*)/*dv*)∙c/(4π*L*), where 1 denotes the refractive index in vacuum, so the vacuum degree in the inner region of the vacuum cell will affect the measurement accuracy. Because the air in the inner space was pumped lower than 0.01 Pa before sealed by use of one turbomolecular vacuum pump, the vacuum degree would introduce a relative measurement uncertainty of less than 1 × 10^−7^ to (N − 1) on the basis of Ciddor’s equation.

To summarize, the 4 error sources mentioned above bring a combined standard uncertainty of 4.7 × 10^−6^ to (N − 1) following the Guide to the Expression of Uncertainty in Measurement (GUM). It can be seen from equation (6) that the measured object in the proposed scheme is (N − 1) rather than N, which brings remarkable merits^40^. We can do the following mathematical transformation: *d*N/N = (N − 1)/N∙*d*(N − 1)/(N − 1), from which we can find that the relative measurement uncertainty of N improves 1/(N − 1) times on equal conditions. Therefore, the combined standard uncertainty of the gas refractivity measurement scheme is (N − 1) × 4.7 × 10^−6^. Under general circumstances, the air refractive index is almost equal to 1.00027 and (N − 1) is nearly 2.7 × 10^−4^, then the combined standard uncertainty of air is 1.3 × 10^−9^.

## Discussion

When making comparisons between the measurement data and computed values by Ciddor’s equation, in addition to the measurement uncertainty of the proposed method, their difference is partly caused by the computed values by Ciddor’s equation. Because the measurement accuracies of these environmental sensors result in an uncertainty of ~4.5 × 10^−8^ to the computed values. Additionally, the response times of these sensors still have influences on the discrepancy, since the proposed measurement apparatus stands on real-time measurement approach while the air refractive index derived from Ciddor’s equation lags behind real-time values. That is to say, because we did not control the environmental conditions severely during the experiment, the environmental parameters would change all the time and the air refractive index would fluctuate on equal size and at equal rate with them, but the slow environmental sensors can’t follow these changes.

In conclusion, we have developed a real-time absolute gas refractometer with a measuring range from 1.0000037 to 1.0375 and a combined standard uncertainty of ~(N − 1) × 4.7 × 10^−6^ (as for air: 1.3 × 10^−9^) at 1530.8 nm using spectral interferometry of frequency comb, resolved by a VIPA-based spectrometer. Because the proposed approach does not need gas-filling, gas-pumping or mechanical scanning, one single measurement only takes 10 ms, which is promising to monitor the gas refractivity with fast and drastic change. Besides, it can be used under conditions without severe control of environmental parameters. Once our self-made movable frequency comb is employed, the gas refractometer will be suitable for *in-situ* monitoring on the spots of industry. If we fill different gases in the chamber, the refractive index of different gases can be measured. As we have mentioned before, the refractivity measurements of N_2_ and He, are important and useful. Besides, the measurement of the CO_2_ refractivity makes great sense not only in computing how much the CO_2_ contributes to the air refractive index but also in monitoring the greenhouse effect. In addition, the refractive indices measurements of liquids and solids are feasible by adjusting the measurement system in existence. Recently, the refractive index measurement of water and CaF_2_ has attracted more and more attention. The reason is that the precise optical characteristics of water and CaF_2_ play a significant role in upgrading the existing semiconductor EUV (extreme ultraviolet) lithography. Due to the improvement of EUV frequency comb^41^, the refractive index of water and CaF_2_ in extreme ultraviolet can be measured accurately. These entire potential advancements mentioned above are in the next step of our research.

## Methods

### Mode filtering

The initial frequency comb with a 250 MHz repetition rate is too densely spaced to be resolved by the VIPA-based spectrometer. Therefore, we utilized a FFP cavity to filter out unwanted comb lines. When using a FP cavity as a mode filter to increase the mode spacing of a frequency comb, the FSR is chosen to be an integer multiple of the initial mode spacing. In our case, the FSR is 5 GHz and only every 20th mode is transmitted through the FFP cavity, while the other ones are rejected. Its length should be stabilized to guarantee FSR = 20*f*_*rep*_. If the cavity is detuned from the optimum length, the increasing mismatch between *f*_*rep*_ and FSR leads to lower transmission of some modes and thus a reduction in the amplitude of successive maxima. Here, we use dither-locking to lock the cavity length to the maximum of the transmission resonance corresponding to twenty times the comb repetition frequency. Specifically, we first generated a 10 kHz dither signal by a waveform generator (Agilent, E8663D). The signal is amplified by a piezoelectric Controller (Throlabs, BPC303) and then sent to the PZT actuator, modulating the optical phase of the frequency comb. Ten percent of the cavity transmission is detected through one photodetector (New Focus, 1811-FC) and then demodulated using a lock-in amplifier (Stanford Research Systems, SR830) synchronizing with the modulation frequency of 10 kHz. As a result, an error signal with zero crossings at cavity transmission peaks is generated. Meanwhile, this error signal is low-pass filtered and fed-back to one proportional-integral controller (PIC, Newport, LB1005) forcing the PZT to stretch a short segment of fiber axially inside the FFP cavity. Finally, the error signal is locked to the zero-crossing point and the cavity length is stabilized to the optimum length.
